# Allogeneic Vγ9Vδ2 T-Cell Therapy Promotes Pulmonary Lesion Repair: An Open-Label, Single-Arm Pilot Study in Patients With Multidrug-Resistant Tuberculosis

**DOI:** 10.3389/fimmu.2021.756495

**Published:** 2021-12-15

**Authors:** Juan Liang, Liang Fu, Man Li, Yuyuan Chen, Yi Wang, Yi Lin, Hailin Zhang, Yan Xu, Linxiu Qin, Juncai Liu, Weiyu Wang, Jianlei Hao, Shuyan Liu, Peize Zhang, Li Lin, Mohammed Alnaggar, Jie Zhou, Lin Zhou, Huixin Guo, Zhaoqin Wang, Lei Liu, Guofang Deng, Guoliang Zhang, Yangzhe Wu, Zhinan Yin

**Affiliations:** ^1^ Zhuhai Institute of Translational Medicine, Zhuhai People’s Hospital (Zhuhai Hospital Affiliated with Jinan University), Jinan University, Zhuhai, China; ^2^ National Clinical Research Center for Infectious Diseases, Guangdong Key Laboratory of Emerging Infectious Diseases, Shenzhen Third People’s Hospital, Southern University of Science and Technology, Shenzhen, China; ^3^ The Biomedical Translational Research Institute, Faculty of Medical Science, Jinan University, Guangzhou, China; ^4^ Beijing Chest Hospital, Capital Medical University, Beijing, China; ^5^ Department for gdT Clinical Research and Development, Guangdong GD Kongming Biotech Ltd., Guangzhou, China; ^6^ Tongji Chibi Hospital, Tongji Medical College, Huazhong University of Science and Technology, Chibi, China; ^7^ Department for Tuberculosis Control, Foshan Fourth People’s Hospital, Foshan, China; ^8^ Department for Tuberculosis Control, Centre for Tuberculosis Control of Guangdong Province, Guangzhou, China

**Keywords:** multidrug-resistant TB, allogeneic Vγ9Vδ2 T cells, immunotherapy, immune regulation, clinical study

## Abstract

The WHO’s “Global tuberculosis report 2020” lists tuberculosis (TB) as one of the leading causes of death globally. Existing anti-TB therapy strategies are far from adequate to meet the End TB Strategy goals set for 2035. Therefore, novel anti-TB therapy protocols are urgently needed. Here, we proposed an allogeneic Vγ9Vδ2 T-cell-based immunotherapy strategy and clinically evaluated its safety and efficacy in patients with multidrug-resistant TB (MDR-TB). Eight patients with MDR-TB were recruited in this open-label, single-arm pilot clinical study. Seven of these patients received allogeneic Vγ9Vδ2 T-cell therapy adjunct with anti-TB drugs in all therapy courses. Cells (1 × 10^8^) were infused per treatment every 2 weeks, with 12 courses of cell therapy conducted for each patient, who were then followed up for 6 months to evaluate the safety and efficacy of cell therapy. The eighth patient initially received four courses of cell infusions, followed by eight courses of cell therapy plus anti-MDR-TB drugs. Clinical examinations, including clinical response, routine blood tests and biochemical indicators, chest CT imaging, immune cell surface markers, body weight, and sputum *Mycobacterium tuberculosis* testing, were conducted. Our study revealed that allogeneic Vγ9Vδ2 T cells are clinically safe for TB therapy. These cells exhibited clinical efficacy in multiple aspects, including promoting the repair of pulmonary lesions, partially improving host immunity, and alleviating *M. tuberculosis* load *in vivo*, regardless of their application in the presence or absence of anti-TB drugs. This pilot study opens a new avenue for anti-TB treatment and exhibits allogeneic Vγ9Vδ2 T cells as promising candidates for developing a novel cell drug for TB immunotherapy.

**Clinical Trial Registration:**

(https://clinicaltrials.gov/ct2/results?cond=&term=NCT03575299&cntry=&state=&city=&dist=) ( NCT03575299).

## Introduction

Tuberculosis (TB), a major chronic infectious disease of the lungs caused by *Mycobacterium tuberculosis*, has become a top killer among infectious diseases due to an epidemic of coinfections and drug resistance (1, 2). According to the World Health Organization’s “Global tuberculosis report 2020,” in 2019, there were approximately two billion individuals infected with *M. tuberculosis* worldwide. These included an estimated 10 million new TB patients and half a million rifampicin-resistant patients, of which 78% are multidrug-resistant (MDR-TB). The treatment of patients with MDR-TB is challenging (3, 4), because of the requirement for long treatment cycles, low efficacy and side effects of anti-TB drugs, poor compliance of patients, easy recurrence, and a susceptibility for development of extensive drug resistance (XDR). The cases of TB drug resistance have greatly postponed the accomplishment of the End TB Strategy set for 2035. Therefore, in addition to traditional anti-TB chemical drugs, breakthroughs in new TB therapies and prevention strategies, such as immune cell-based biotherapies or vaccines, are urgently needed to accelerate the annual decline in the global TB incidence rate by approximately 17% in 2025 and per year afterward. Accomplishing these goals would then hopefully control the TB epidemic.

In the past, cell therapies, including stem cells (5, 6), αβ T cells (7, 8), NK cells (9), and γδ T cells (10), have been extensively utilized in the treatment of tumor- or autoimmune-related diseases, demonstrating sound clinical efficacy. Nonetheless, research on immune cell therapy in the field of infectious diseases, specifically *M. tuberculosis* infection, has been very limited (11, 12). As the pathogenesis of TB has been closely correlated with suppressive immune functions of the host, immune cell therapy should be a new promising direction for controlling TB. In particular, γδ T cells, a subset of T lymphocytes that bridges innate and adaptive immunity (13), account for less than 10% of T lymphocytes, with the Vγ9Vδ2 cell subset being dominant in peripheral blood.

Using a non-human primate model, the group of Chen proposed Vγ9Vδ2 T-cell-based cell therapy for the treatment of TB (11, 12). This groundbreaking study exhibited that adoptive cell transfer therapy of pre-expanded autologous Vγ9Vδ2 T cells from peripheral blood mononuclear cells (PBMCs) before *M. tuberculosis* infection could significantly decrease the postinfectious *M. tuberculosis* burden in the liver, kidneys, and spleen of macaques. Importantly, the study showed the remarkable alleviation of lesions in the lung tissues of infected animals, indicating the clinical prospect of Vγ9Vδ2 T cells for the treatment of patients with TB. However, the clinical efficacy of Vγ9Vδ2 T cells against chronic *M. tuberculosis* infections of patients with TB remains to be addressed. Furthermore, autologous Vγ9Vδ2 T cells of patients with TB are functionally impaired and therefore are quite difficult to be expanded *ex vivo*. In this context, allogeneic Vγ9Vδ2 T-cell-based immunotherapy (10, 14) should be an optimal strategy for the treatment of patients with TB and even MDR-TB.

In the present study, we utilized allogeneic Vγ9Vδ2 T cells for the treatment of patients with MDR-TB who had significantly limited options in effective anti-TB drugs. We enrolled eight patients with MDR-TB to receive allogeneic Vγ9Vδ2 T-cell therapy, and conducted a total of 12 cell transfer infusions for each patient. We found that allogeneic Vγ9Vδ2 T cells exhibited promising safety in patients with MDR-TB. Most importantly, we observed a significant relief in pulmonary lesions in all eight patients receiving either allogeneic Vγ9Vδ2 T-cell treatment alone or in combination with anti-MDR-TB drugs. This is a clear evidence of the promising efficacy of the therapy. Collectively, allogeneic Vγ9Vδ2 T-cell-based immunotherapy may provide a novel therapeutic strategy for patients with MDR-TB, and future extensive clinical application of allogeneic Vγ9Vδ2 T cells may be beneficial for controlling TB epidemics worldwide.

## Materials and Methods

### Enrollment of Patients With MDR-TB and Ethics

Between July and December 2018, eight patients with MDR-TB diagnosed using *M. tuberculosis* sputum smear microscopic examination, culture, and drug sensitivity testing were recruited in our study after signing an informed consent. The age of the patients ranged between 18 and 50 years old. Patients with other conditions, including HIV, hepatitis B, diabetes, tumor, hypertension, coronary heart disease, endocrine system disease, mental disease, neurological disease, vascular circulation system disease, or other disease, were excluded from our study.

The Regional Ethics Committee of Shenzhen Third People’s Hospital approved the study protocol (Approval ID SZLY2018017). All participants provided signed written informed consents in accordance with the Declaration of Helsinki. This study has been registered in the Clinical Trials website (ClinicalTrials.gov, ID: NCT03575299).

### *Ex Vivo* Selective Expansion of Vγ9Vδ2 T Cells From PBMCs of Healthy Donors

First, we obtained 100 ml peripheral blood from a healthy donor who had received and passed the screening of infectious diseases, including hepatitis B, hepatitis C, syphilis, and HIV. Human PBMCs were isolated using Ficoll–Paque-based density gradient centrifugation. Next, we induced and extended the population of Vγ9Vδ2 T cells using our patented formula (Patent No. ZL201811580040.2). PBMCs at a density of 3 × 10^6^ cells/ml were cultured in RPMI 1640 (Gibco) containing 10% FBS and 1% penicillin–streptomycin and supplemented with 50 μM zoledronic acid (Sigma), 100 IU/ml recombinant human interleukin (IL)-2 (Beijing Four Rings Bio-Pharm Co.), 100 IU/ml recombinant human IL-15 (Peprotech), and 70 μM L-ascorbic acid (Sigma) on day 0. Afterwards, fresh medium and cytokines were replaced every 2–3 days. After being cultured for approximately 10 days, the viability of Vγ9Vδ2 T cells was determined using flow cytometry utilizing the PE-annexin V apoptosis kit (BD Biosciences, San Jose, CA, USA). In addition, Vγ9Vδ2 T cells were stained using anti-human CD3-APC-H7 (clone SK7; BD Biosciences), anti-human TCR Vδ2-Percp (clone B6; Biolegend), and anti-human CD314 (NKG2D)-PerCP/Cy5.5 (clone 1D11; BD Biosciences) antibodies to identify the cell phenotypes.

### Adoptive Transfer Therapy of MDR-TB Using Allogeneic Vγ9Vδ2 T Cells

During the process of cell expansion, cells were tested twice for pathogens, including bacteria, fungi, mycoplasma, and endotoxins. Vγ9Vδ2 T cells adhering to the quality criteria, including contamination-free, proportion higher than 90%, and activity greater than 90%, were used for adoptive transfer to patients. On the day of cell infusion, cells were washed three times with normal saline solution. Next, 1 × 10^8^ cells were placed in 100 ml normal saline solution containing 1% human serum albumin. Subsequently, cells were infused in patients within half an hour. Each of the eight patients received each infusion of Vγ9Vδ2 T cells from a different donor.

### Immunophenotype Analysis for the Evaluation of Alterations in Peripheral Immune Cell Functions

We collected 5 ml of peripheral blood from patients before Vγ9Vδ2 T-cell infusion. The routine protocols of our lab were used for the isolation of PBMCs from the blood. The phenotype of immune cells, including T cells, NK cells, γδ T cells, B cells, and their subsets, was analyzed using flow cytometry (FACSanto™ II; BD Biosciences). The antibodies used included the following: PerCP-CY5.5-conjugated anti-CD3, anti-CD8, anti-CD19, and anti-CD94; APC-H7-conjugated anti-CD3, anti-CD4, and anti-CD45; FITC-conjugated anti-CD45RA and anti-CD8; BV510-conjugated anti-CD8 and anti-CD196; PE-conjugated anti-HLA-DR, anti-KIR, and anti-Vδ2; PE-CY7-conjugated anti-CD4 and anti-NKG2D; BV421-conjugated anti-CD56, anti-27, anti-CD194, anti-TCRγδ, and anti-P30; BV510-conjugated anti-CD8 and anti-CD196; Alexa Fluor 647-conjugated anti-CCR7, anti-CXCR5, anti-PD-1, and anti-CD186; and Alexa Fluor 484-conjugated anti-CD183. All antibodies were purchased from BD Biosciences.

### Clinical Evaluation of the Therapeutic Efficacy of Allogeneic Vγ9Vδ2 T Cells

To evaluate the therapeutic efficacy, all patients underwent chest CT examination before Vγ9Vδ2 T-cell infusion and every 2 months afterwards. The body weight of patients was also recorded before each Vγ9Vδ2 T-cell infusion and again in each follow-up. Routine blood and biochemical examinations were also performed before each Vγ9Vδ2 T-cell infusion. One of the gold standards in the treatment of TB is to change positive *M. tuberculosis* sputum to negative. Therefore, we conducted sputum tuberculosis examinations, including sputum acid-fast staining and sputum *M. tuberculosis* culture throughout this clinical study.

Furthermore, we performed plasma metabolite detection using plasma samples from patient #8 who only received allogeneic Vγ9Vδ2 T-cell therapy (first 4 times). To accomplish this, 5 ml of peripheral blood was collected from patient #8 before each Vγ9Vδ2 T-cell infusion, and metabolites were detected in the plasma using mass spectrometry (Guangdong Longsee Biomedical Co. Ltd.).

Two stool samples were collected from patient #8, one before and one after receiving allogeneic Vγ9Vδ2 T-cell therapy using the Fecal Microbial Genome Protective Kit (LS-R-P-003, Longsee). Microbial DNA was extracted using the Stool Microbial Genomic DNA Extraction Kit (LS-R-N-015, Longsee) in accordance with the instructions of the manufacturer. The V3–V4 highly variable region of 16S rRNA was amplified using PCR with primers 338F: 5′-ACTCCTACGGGAGGCAGCA-3′ and 806R: 5′-GGACTACHVGGGTWTCTAAT-3′. PCR amplification products were detected using 2% agarose gel electrophoresis. Subsequently, target fragments were cut and recovered using the AxyPrep DNA Gel Recovery Kit (AP-GX-50G, Axygen). Fluorescence quantification of the recovered products was performed using PCR amplification with the Quant-iT PicoGreen dsDNA assay kit (P11496, Invitrogen) in a FLx800 microplate reader (BioTek). After serially diluting each qualified sequencing library (index sequence was not repeatable), they were mixed in corresponding proportion according to the required sequencing volume and then denatured to a single strand by NaOH for sequencing. The MiSeq Reagent Kit V3 (600 cycles; MS-102-3003, Illumina) was used for sequencing. The MiSeq-PE250 sequencer was used for 2 × 300 bp paired-end sequencing on an Illumina platform according to standard protocols.

### Statistical Analysis

Data were analyzed using *t*-test with GraphPad Prism 7.0 software. *P*-values of <0.05 were considered statistically significant.

## Results

### Research Cohort and Clinical Immunotherapy Protocol

Between July and December 2018, we enrolled eight patients with MDR-TB in our study. The information of these eight patients as well as drug treatment protocols is shown in **Table 1**. All enrolled patients were diagnosed with MDR-TB. Rather than a fixed drug recipe, the drug protocol for the treatment of TB was determined by a group of physicians according to the clinical symptoms of individual patients. Each patient received 12 cycles of adoptive transfer cell therapy. Therefore, the eight patients with MDR-TB received a total of 96 cell infusions in this clinical study. In particular, patient #8 only received allogeneic Vγ9Vδ2 T-cell therapy in the first 4 of the 12 total courses according to the requirement of the patient and continued with anti-TB drugs. The medical record of patient #8, which clearly illustrates the developmental timeline from TB to MDR-TB, is shown in **Supplementary Table 1**. The brief therapeutic protocol of this patient is shown in **Supplementary Figure 1**.

**Table 1:** Preliminary information of the eight patients.

	Gender		Age, years	Diagnosis	Case definition	Drug regimen	Times of cell therapy	Cell dosage
#1	Female	23		MDR-TB	Retreatment	PZA, Mfx, Cs, Pto, Am	12	~1 × 10^8^
#2	Male	42		MDR-TB	Retreatment	Lzd, Mfx, Cs, Cfz	12	~1 × 10^8^
#3	Male	47		MDR-TB	Retreatment	Lzd, Mfx, Cs, Cfz, Pto	12	~1 × 10^8^
#4	Male	39		MDR-TB	Retreatment	Mfx, Cs, Cfz, PAS	12	~1 × 10^8^
#5	Female	24		MDR-TB	Retreatment	Lzd, Cs, Mfx, PZA, Am	12	~1 × 10^8^
#6	Male	50		MDR-TB	Retreatment	Lzd, Cs, Mfx, PZA, Am	12	~1 × 10^8^
#7	Male	34		MDR-TB	Retreatment	Lfx, Am, INH, PZA, EMB	12	~1 × 10^8^
#8	Male	41		MDR-TB	Retreatment	*	12	~1 × 10^8^

MDR-TB, multidrug-resistant tuberculosis; Am, amikacin; Cfz, clofazimine; Cs, cycloserine; EMB, ethambutol; INH, isoniazid; Lzd, linezolid; Mfx, moxifloxacin; PAS, p-aminosalicylic acid; PZA, pyrazinamide; Pto, protionamide.

*Only Vγ9Vδ2 T cells were used for treatment in four courses of therapy according to the requirement of the patient. Thereafter, anti-TB drugs (Lzd, Mfx, Cs, Cfz, propionyl isoniazid) were used.

**Figure 1:**
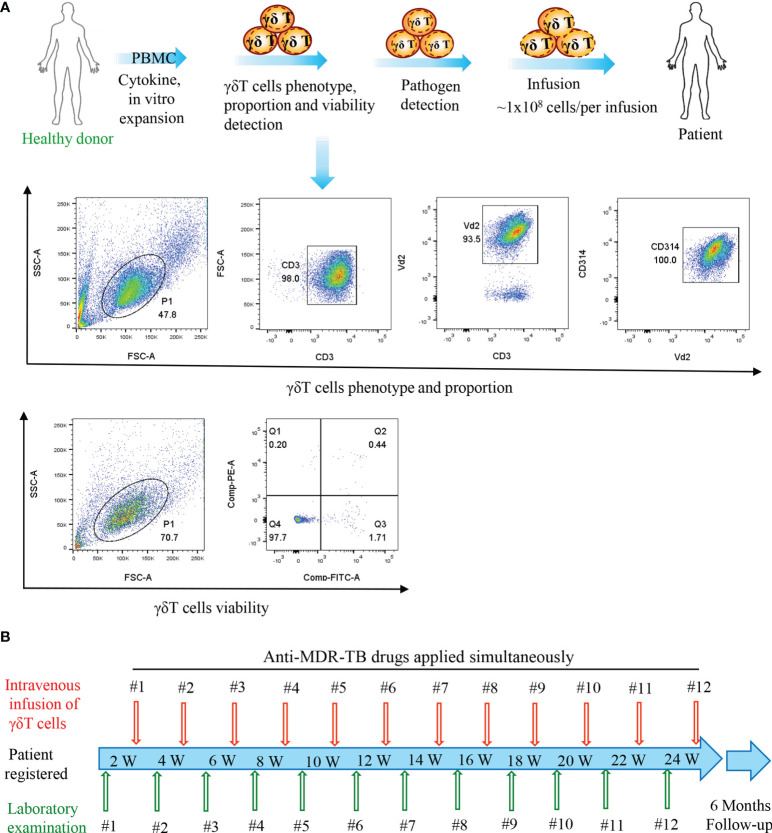
Flow diagram of the treatment of patients. **(A)** Vγ9Vδ2 T-cell expansion *in vitro*, quality control, and infusion. **(B)** Time points of Vγ9Vδ2 T-cell infusion and laboratory examination.

Regarding the therapy protocol (**Figure 1**), we *ex vivo* expanded cells in the peripheral blood collected from healthy donors using our previously established methodology (10, 14). We adoptively infused the expanded cells into the enrolled patients after they passed quality control requirements, which included purity (≥90%), viability (≥90%), and lack of pathogens (**Figure 1A**). In our protocol, patients received a single treatment of 1 × 10^8^ Vγ9Vδ2 T cells every half month with a total of 12 courses completed in 6 months, followed by a 6-month follow-up (**Figure 1B**). Notably, all examinations including routine blood and biochemical assays, immune function phenotype, and chest CT were performed at the scheduled time points.

### Infusions of Allogeneic Vγ9Vδ2 T Cells Were Safe and Did Not Reduce the Side Effects of Anti-MDR-TB Drugs

**Figure 2:**
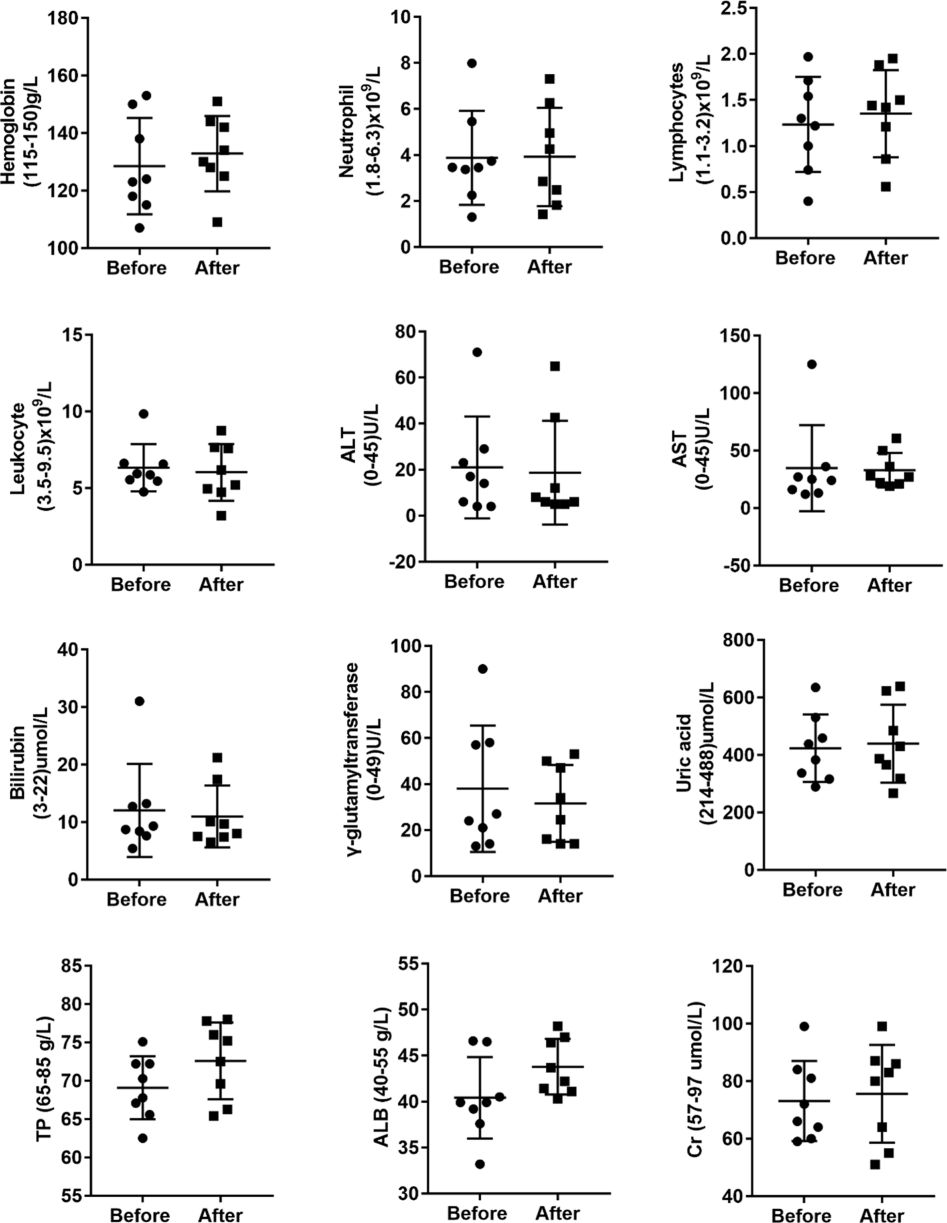
Allogeneic Vγ9Vδ2 T-cell therapy did not statistically reduce the clinical side effects induced by the application of anti-MDR-TB drugs. Side effects investigated before and after cell therapy included changes in hemoglobin, neutrophils, lymphocytes, leukocytes, alanine aminotransferase (ALT), aspartate aminotransferase (AST), bilirubin, γ-glutamytransferase, uric acid, total protein (TP), albumin (ALB), and creatinine (Cr) levels.

Given that anti-TB drugs have various side effects, we explored whether these adverse effects were minimized under allogeneic Vγ9Vδ2 T-cell treatment. We statistically compared the alterations in 12 immunological/biochemical parameters, namely, hemoglobin, neutrophils, lymphocytes, leukocytes, alanine aminotransferase, aspartate aminotransferase, bilirubin, γ-glutamytransferase, uric acid, total protein, albumin, and creatinine. All of the biochemical markers are routinely and adversely changed by anti-TB drugs (**Figure 2**), before and after the 12 courses of cell therapy. Adverse effects were defined according to the Common Terminology Criteria for Adverse Events version 4.0. We observed that allogeneic Vγ9Vδ2 T-cell therapy did not significantly reduce these 12 types of adverse effects. Moreover, this finding also indicated that allogeneic Vγ9Vδ2 T-cell therapy does not exert additional unexpected side effects, thereby demonstrating its promising clinical safety, as supported by the 96 total courses of cell treatment in this trial study (**Table 1**).

**Figure 3:**
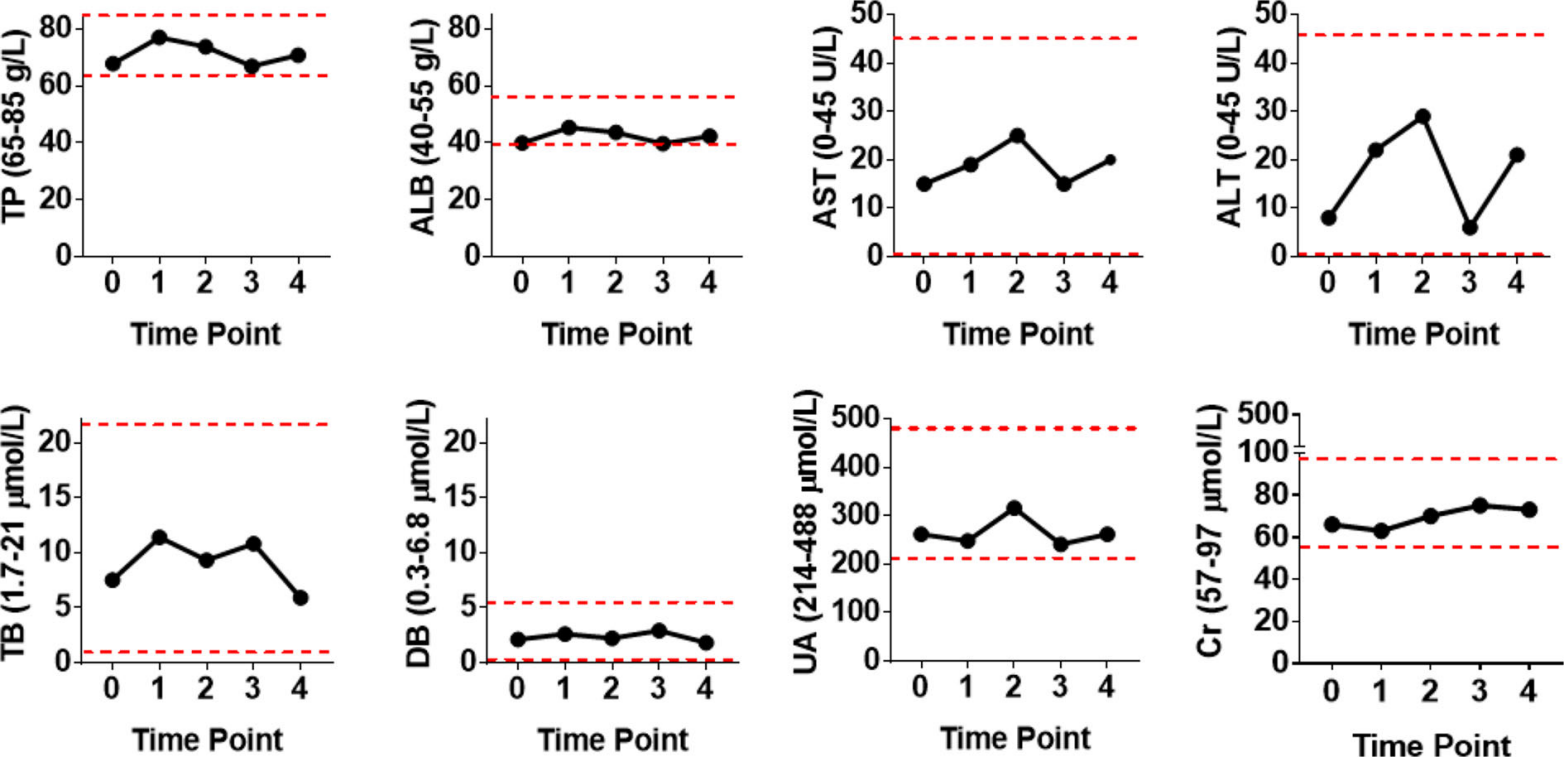
Blood biochemical examinations of patient #8 before and after receiving four courses of Vγ9Vδ2 T-cell immunotherapy. Assayed biochemical markers included total protein (TP), albumin (ALB), total bilirubin (TB), direct bilirubin (DB), alanine transaminase (ALT), aspartate transaminase (AST), uric acid (UA), and creatinine (Cr) levels. Red dashed lines stand for the normal reference range widely used in the clinical setting.

Because patient #8 only received cell infusion exclusively in the first 4 out of the 12 total courses, we conducted intensive analyses to compare the biochemical alterations before and after the Vγ9Vδ2 T-cell therapy. We assessed various serological parameters, including total protein, albumin, total bilirubin, direct bilirubin, alanine transaminase, aspartate transaminase, creatinine, and uric acid. We found that all these biochemical markers were maintained at a normal level following Vγ9Vδ2 T-cell infusion (**Figure 3**). This suggests that allogeneic Vγ9Vδ2 T-cell therapy did not cause any impairments in liver and kidney functions, further indicating the clinical safety of allogeneic Vγ9Vδ2 T cells.

### Allogeneic Vγ9Vδ2 T-Cell Therapy Effectively Improved Pulmonary Lesions

**Figure 4:**
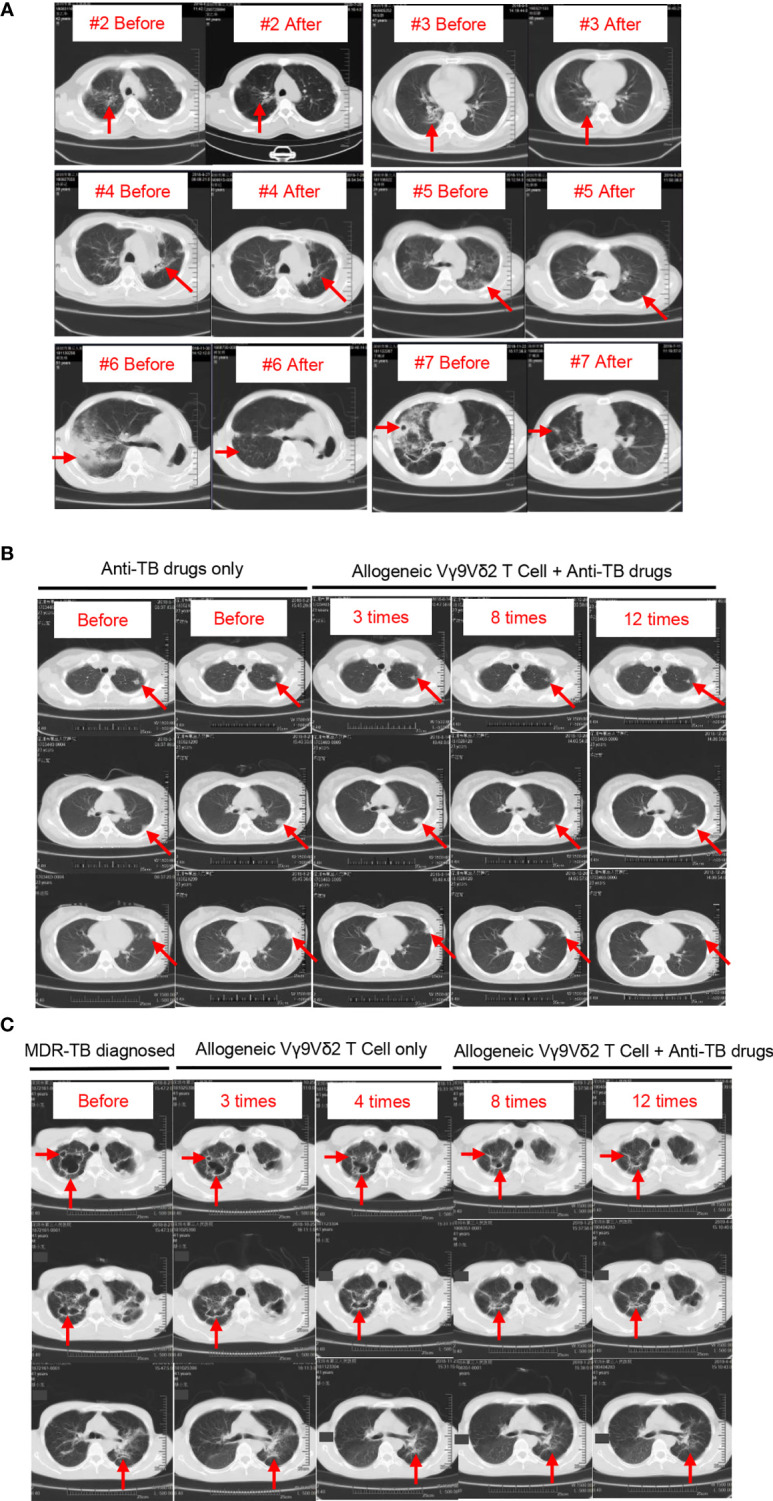
Chest CT images of the eight patients with MDR-TB before and after treatment. Pathological sites are indicated by red arrows. **(A)** Chest CT images of patients #2 through #6. After enrollment, these patients received Vγ9Vδ2 T-cell infusions and treatment with anti-TB drugs. CT images of two time points, one before and one after treatment. **(B)** Chest CT images of patient #2. This patient received anti-TB drugs before enrollment, and CT images show the progressive deterioration in lesion locations (first and second columns). After enrollment, this patient continued to receive anti-TB drugs (drug recipe not changed) plus Vγ9Vδ2 T-cell infusions. The size of pathological sites was gradually reduced with the progress of cell therapy (third through fifth columns). **(C)** Chest CT images of patient #8. The progressive repair of the cavitary lesion in pulmonary pathological sites after four courses of cell therapy alone (first through third columns) is shown. In the case of combined treatments with Vγ9Vδ2 T cells plus anti-MDR-TB drugs, pulmonary lesions continued to decrease (fourth and fifth columns).

All patients in our study received Vγ9Vδ2 T-cell infusions and concurrent anti-MDR-TB medications. We observed that lung lesions were reduced in all patients after the end of treatment (**Figure 4**). In particular, we noticed that the lung lesions were significantly decreased at the end of treatment compared with those before treatment in patients #2 through #6 (**Figure 4A**). Due to the simultaneous administration of Vγ9Vδ2 T cells and anti-TB drugs, it was difficult to estimate the efficacy of Vγ9Vδ2 T cells. The observed reduction in the lung lesions of patients might be attributed either to the anti-TB drugs, Vγ9Vδ2 T cells, or the synergism of both. However, we speculated that Vγ9Vδ2 T cells might have promoted the improvement of lung lesions.

Next, we evaluated the clinical status of two other patients to confirm our speculation. Patient #1 who received anti-TB treatment before Vγ9Vδ2 T-cell infusions showed progressive deterioration in lesion locations, as indicated by the red arrows in **Figure 4B** (first and second columns). We specifically found that the administration of allogeneic Vγ9Vδ2 T cells inhibited the progress of lesion locations during the process of the 12 courses of therapy, as indicated in **Figure 4B** (third through fifth columns). This finding endorsed the promising clinical efficacy of allogeneic Vγ9Vδ2 T-cell-based immunotherapy for MDR-TB and was further supported by the more convincing therapeutic effect seen in patient #8. According to contrast-enhanced axial CT images of the chest, patient #8 had severe cavitary pulmonary lesions, as indicated by the red arrows in **Figure 4C**. Cavitary lesions have a rough and discontinuous inner margin, implying the active state of *M. tuberculosis* that induced them. After three and four courses of cell therapy, we noticed that the size of the cavitary lesions was gradually and significantly reduced (second and third columns; **Figure 4C**), suggesting an observable therapeutic efficacy of allogeneic Vγ9Vδ2 T cells. Consecutively, both Vγ9Vδ2 T cells and anti-MDR-TB drugs were simultaneously administered in enrolled patients. Accordingly, we noticed the synergistic effects on the improvement of cavitary lesion or lesion locations of such a combined therapy strategy (fourth and fifth columns; **Figure 4C**).

### Allogeneic Vγ9Vδ2 T Cells Enhanced Some Immune Functions of PatientsWith MDR-TB

**Figure 5:**
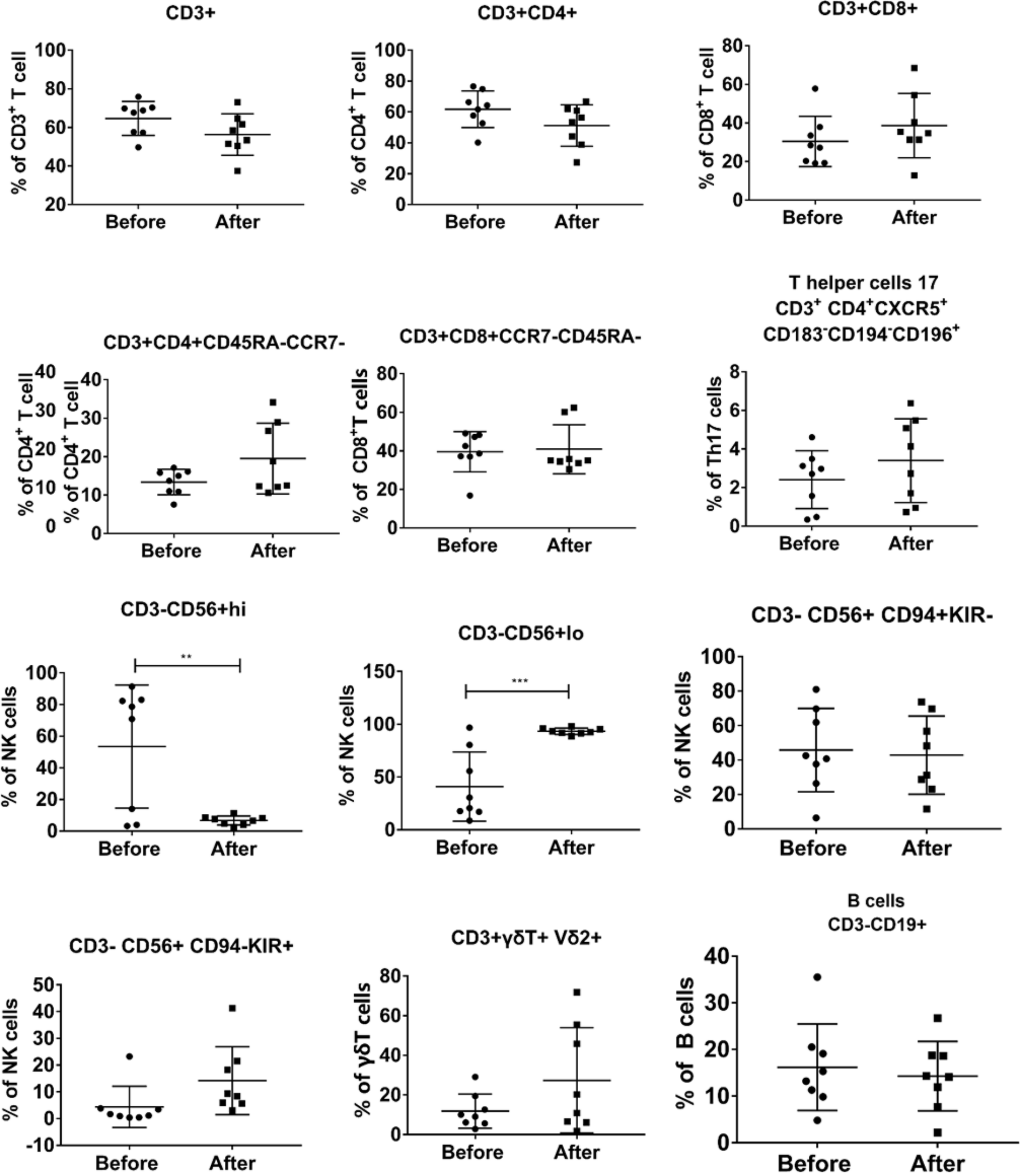
Evaluation of the changes in the phenotype of peripheral blood immune cells before and after Vγ9Vδ2 T-cell immunotherapy in the eight patients. PBMCs were collected from all patients before and after Vγ9Vδ2 T-cell infusions. Immunological phenotypes, which were analyzed using flow cytometry, included CD3^+^ T cells, CD4^+^ T cells, CD8^+^ T cells, NK cells, γδ T cells, B cells, and their subsets.

Because previously Vγ9Vδ2 T cells have been demonstrated to regulate the immune function of αβ T cells, NK cells, B cells, and other cells *in vivo* (10, 14, 15), it was crucial to analyze the immune phenotypes of all patients before and after the infusion of Vγ9Vδ2 T cells (**Figure 5**). We found that the percentages of CD3^+^, CD4^+^, CD8^+^, γδ T cells, B cells, and some cell subsets were not statistically different at the end of treatment compared with those before treatment. Conversely, the proportion of immature NK cells that were CD3^−^CD56^+^hi was significantly reduced with a concomitant significant increase in the proportion of mature NK cells that were CD3^−^CD56^+^lo. The data suggested that allogeneic Vγ9Vδ2 T-cell therapy partially enhanced host immune functions by regulating the proportion of mature NK cells.

**Figure 6:**
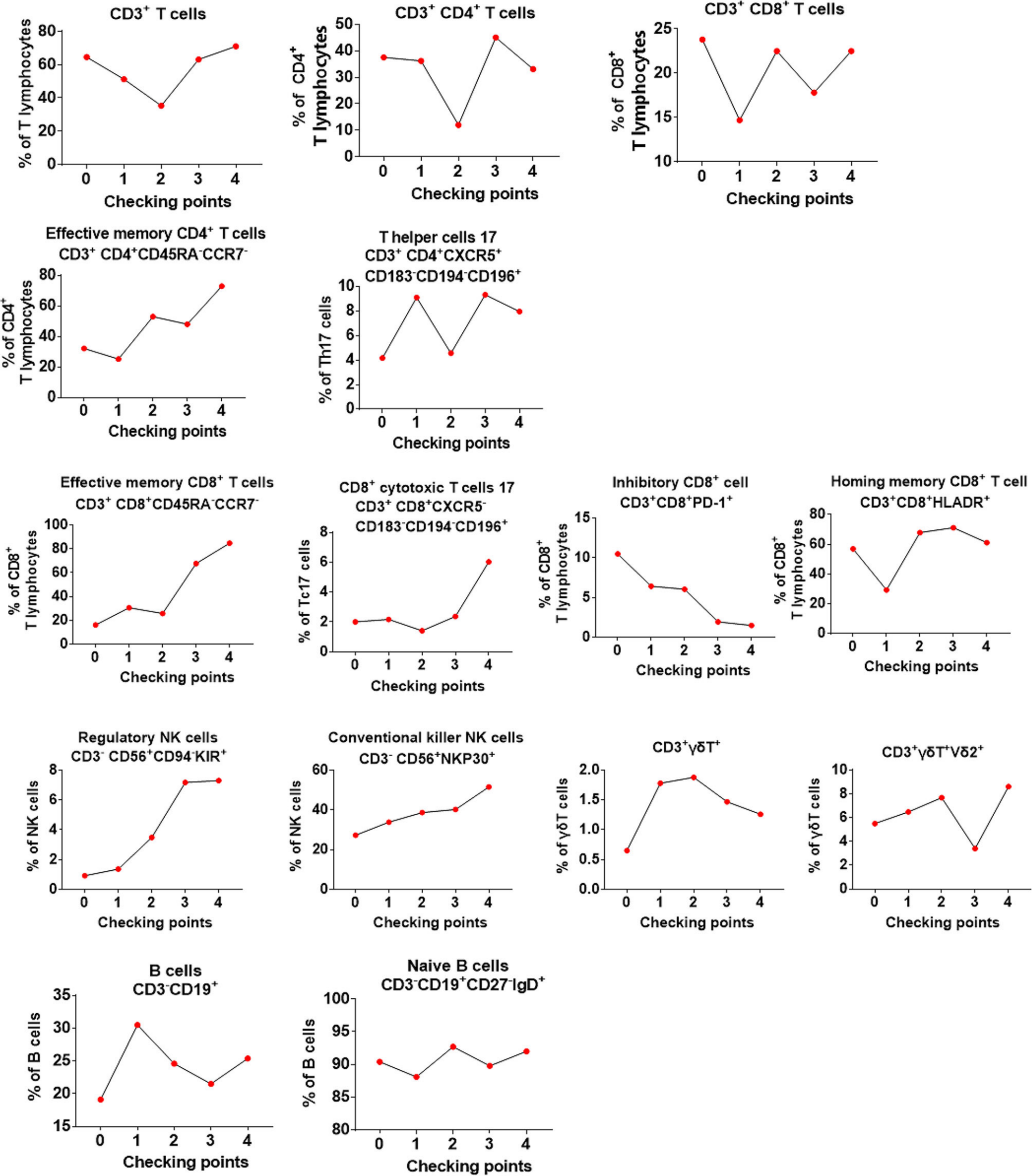
Evaluation of the changes in the phenotype of peripheral blood immune cells for the first 4 courses of Vγ9Vδ2 T-cell therapy in patient #8. In the graphs, point “0” indicates the immunophenotype before Vγ9Vδ2 T-cell treatment, whereas “1–4” indicates the immunophenotype from the first time to the fourth time of cell treatment.

We also analyzed the immune phenotypes of patient #8 before and after his limited Vγ9Vδ2 T-cell therapy (4 courses instead of 12; **Figure 6**). We found that although the percentage of CD3^+^, CD4^+^, CD8^+^ T cells, total B cells, and naive B cells (CD3^+^CD19^+^CD27^−^IgD^+^) varied, it remained relatively stable during the courses of cell therapy. By contrast, we observed that the proportion of effective memory CD4^+^ T cells (CD3^+^CD4^+^CD45RA^−^CCR7^−^), effective memory CD8^+^ T cells (CD3^+^CD8^+^CD45RA^−^CCR7^−^), helper T-cell subsets (CD3^+^CD4^+^CXCR5^−^CD183^−^CD194^−^CD196^+^), cytotoxic CD8^+^ T cells (CD3^+^CD8^+^CXCR5^−^CD183^−^CD194^−^CD196^+^), homing memory CD8^+^ T cells (CD3^+^CD8^+^HLADR^+^), and NK cells (CD3^−^CD56^+^CD94^−^KIR^+^ and CD3^−^CD56^+^NKP30^+^) was increased. Notably, the proportion of PD-1^+^CD8^+^ T cells (CD3^+^CD8^+^PD-1^+^) was significantly decreased after cell therapy. As for γδ T cells, we noticed that the proportion of both total and Vδ2^+^ γδ T cells was maintained at a relatively high level following the γδ T-cell infusions. These results indicated that allogeneic Vγ9Vδ2 T-cell therapy enhanced some immune functions by regulating the proportion of αβ T cells and NK cells.

### Allogeneic Vγ9Vδ2 T-Cell Therapy Potentially Benefited Body Weight Gain and Gut Microbiota Regulation

**Figure 7:**
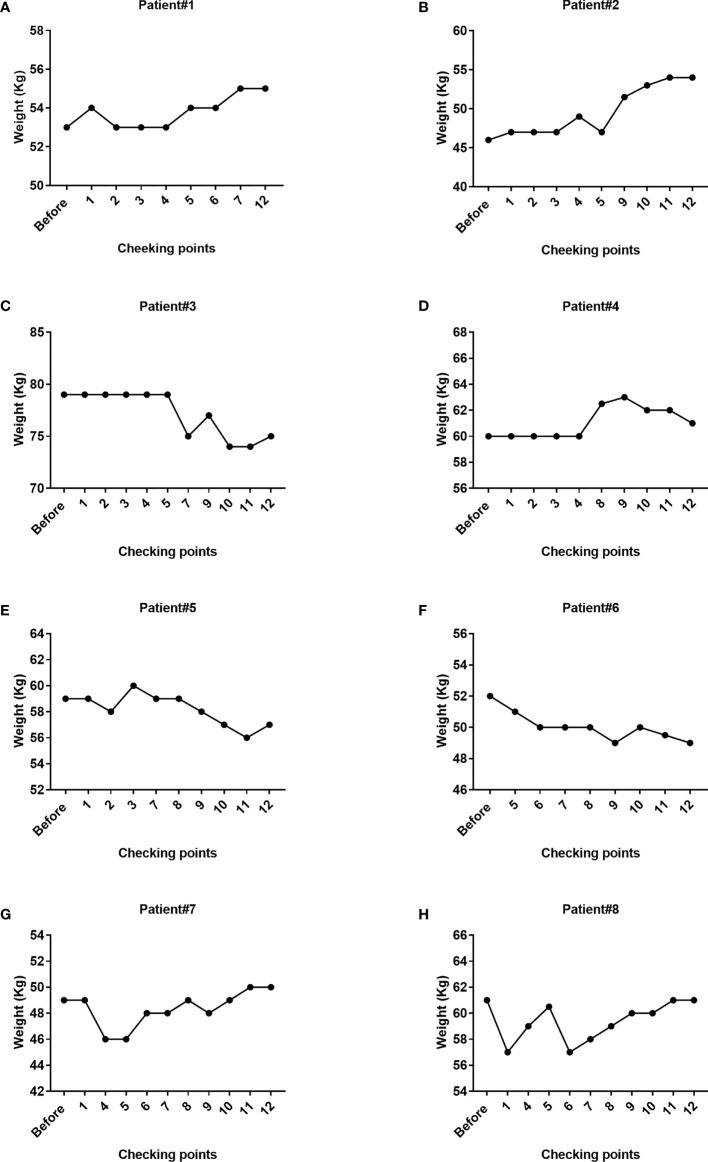
Changes in the body weight of the eight patients during Vγ9Vδ2 T-cell immunotherapy. The weight of patient #1, patient #2, patient #7, and patient #8 steadily increased, whereas the weight of patient #3, patient #5, and patient #6 decreased. The overall trend in patient #4 was a tendency for an increase in body weight. The weight of partial patients was not available at the designated checking points due to missing weight assessments.

Loss of body weight is one of the clinical manifestations in patients with TB. Accordingly, we evaluated the changes in the body weight of all patients before and after cell therapy (**Figure 7**). We specifically observed that 50% of patients had gained weight, whereas 50% exhibited weight loss. Because both the drugging regimen and nutritional status varied across patients, it was difficult to estimate the specific effect of Vγ9Vδ2 T cells in the changes in the overall body weight of patients. Interestingly, we observed a body weight increase from 46 to 49 kg in patient #8 after only four courses of Vγ9Vδ2 T-cell therapy (**Supplementary Figure 2A**). This suggested that cell therapy might improve the overall physical condition of patients. In addition, the metabolite assay in patient #8 revealed that the levels of three carnitine components in blood plasma, which are associated with fatty acid metabolism, namely, decanoyl L-carnitine (2.6–3.8-fold), octanoyl carnitine (1.9–3.2-fold), and dodecanoyl carnitine (1.7–2.6-fold), were greatly reduced (**Supplementary Figures 2B–D**). These results implied that cell therapy might increase the body weight of patients by altering carnitine metabolism.

In the past, gut microbiota have been increasingly associated with host recovery, prevention of *M. tuberculosis* infection, and resistance or clinical efficacy of anti-TB drugs (16–20). Therefore, we investigated whether the limited and only Vγ9Vδ2 T-cell therapy in patient #8 would alter the composition of gut microbiota. We collected two stool samples from patient #8 before and after cell therapy and analyzed them using 16S rRNA gene sequencing. We found increases in 10 types of commensal bacteria or probiotics, whereas the population of seven types of pathogenic bacteria was decreased (**Supplementary Figure 3**). This finding implied that allogeneic Vγ9Vδ2 T-cell therapy might also regulate gut immunity by altering the composition of intestinal microbiota, eventually benefiting the therapeutic efficacy of cell therapy in the patient.

### Allogeneic Vγ9Vδ2 T-Cell Therapy Contributes in Alleviating the *Mycobacterium tuberculosis* Load *In Vivo*

As the *M. tuberculosis* load is a key indicator for evaluating therapeutic efficacy, we performed sputum *M. tuberculosis* detection in all patients (**Table 2**). We observed that *M. tuberculosis* was detected in the sputum of patients regardless of the methods used (sputum smear acid-fast bacillus testing or sputum culture testing) before Vγ9Vδ2 T-cell therapy. After all patients had finished therapy, patient #2, who had severe MDR-TB, still had a *M. tuberculosis-*positive sputum culture. However, the lesions of patient #2 were significantly decreased. The sputum *M. tuberculosis* turned to be negative 3 times consecutively in the remaining seven patients. According to the criteria of the World Health Organization, a 3 times consecutive negative detection of sputum *M. tuberculosis* indicates complete clinical control of *M. tuberculosis* infection. Although we could not determine which component (Vγ9Vδ2 T cells or drugs) played the critical role against *M. tuberculosis*, the cure prevalence proportions were up to 87.5% when patients were treated with Vγ9Vδ2 T cells plus anti-MDR-TB drugs. According to the “Global tuberculosis report 2020” of the World Health Organization, the cure prevalence proportions were 54% when patients were treated with only anti-MDR-TB drugs. Obviously, the results demonstrate that allogeneic Vγ9Vδ2 T-cell therapy contribute in alleviating the *M. tuberculosis* load *in vivo*.

**Table 2:** Sputum *Mycobacterium tuberculosis* examination of all patients before and after Vγ9Vδ2 T-cell immunotherapy.

		#1	#2	#3	#4	#5	#6	#7	#8
Sputum smear (acid-fast bacillus)	Before	−	+	−	+	−	+	+	+
Sputum *M. tuberculosis* culture	Before	+	−	+	+	+	+	+	+
Sputum smear (acid-fast bacillus)	After	−	−	−	−	−	−	−	−
Sputum *M. tuberculosis* culture	After	−	+	−	−	−	−	−	−

For sputum acid-fast bacillus smear examination, “−” means M. tuberculosis was not detected in 300 consecutive fields under the microscope (×1,000), whereas “+” means 3 to 9 M. tuberculosis were found in 100 consecutive fields under the microscope (×1,000). Regarding the sputum culture method, “+” indicates the presence of M. tuberculosis in the sputum.

## Discussion

At present, drug resistance is the most serious challenge for the treatment of tuberculosis. To address this issue, the development of new antituberculosis drugs is urgently needed. Cellular immunotherapy is a new and promising clinical treatment approach. Currently, almost all types of immune cells, such as CD4^+^ T cells, CD8^+^ T cells, NK cells, B cells, myeloid cells, and γδ T cells, have been used as a cellular platform for the development of immunotherapies for various diseases. Among these immune cell candidates, γδ T cells have been increasingly recognized as a new strategic tool for immunotherapy of both tumor and infectious diseases (10, 14, 15, 21–23).

Of note, γδ T cells, specifically the Vγ9Vδ2 subset explored in this study, are unconventional innate immune T cells, which play a significant role in controlling diverse diseases, including bacterial and viral infections, as well as tumorigenesis. Regarding tuberculosis, several published studies have demonstrated a significant reduction in the population of Vγ9Vδ2 T cells in the peripheral blood and alveolar lavage fluid of patients with active pulmonary tuberculosis, indicating that Vγ9Vδ2 T cells play a protective role in tuberculosis (24, 25). Previous studies have also shown that Vγ9Vδ2 T cells recognize *M. tuberculosis*-derived phosphoantigens and 6-O-methylglucose-containing lipopolysaccharides, which, in turn, activate and expand the population of Vγ9Vδ2 T cells (26–29). Activated Vγ9Vδ2 T cells contribute to both innate and adaptive immune responses *via* the secretion of cytokines and the release of cytolytic effectors. In general, Vγ9Vδ2 T cells produce cytokines such as interferon-γ, tumor necrosis factor-α, and IL-17 to enhance the protection against tuberculosis (30–36). Moreover, Vγ9Vδ2 T cells also produce cytolytic effector molecules such as perforin, granzyme B, and granulysin to help kill or inhibit intracellular and extracellular *M. tuberculosis* (12, 27, 32, 37–39). In the *M. tuberculosis*-infected macaque model, the histopathologic changes in pulmonary and extrapulmonary tissues were significantly attenuated by the adoptive transfer of autologous Vγ2Vδ2 T cells (Vγ9Vδ2 T cells) (11). A recent study reported that Vγ2Vδ2 T cells that were selectively immunized with HMBPP-producing attenuated *Listeria monocytogenes* (Lm ΔactA prfA*) in a rhesus TB model triggered rapid responses and long-lasting memory-like responses while amplifying the responses of other T-cell subsets (12). The result was a significant decrease in lung pathological indications and *M. tuberculosis* extrapulmonary dissemination.

Tuberculosis is a chronic infectious disease that usually does not show obvious symptoms until the total compromise of host immune homeostasis. Therefore, targeting immunoregulatory pathways might be a new strategy of anti-TB treatment. Given the reduction in the function of autologous Vγ9Vδ2 T cells and their unique property of targeting infected cells through MHC-unrestricted mechanisms without inducing graft-versus-host disease (40), we innovatively used allogenic Vγ9Vδ2 T cells to treat patients with MDR-TB in our study based on their previously demonstrated clinical safety (10, 14). According to our clinical observations, a total of 96 courses of allogenic Vγ9Vδ2 T-cell infusions in eight patients with MDR-TB exhibited no serious adverse events. In particular, four courses of allogenic Vγ9Vδ2 T cells alone were sufficient to treat patient #8 without causing any liver or kidney function impairment. These results indicated the clinical safety of allogenic Vγ9Vδ2 T cells in patients with TB. The results of the present study along with those of previously published studies (10, 14) have collectively demonstrated that allogenic Vγ9Vδ2 T cells are safe and could be efficiently used in clinical practice.

Tuberculosis is usually accompanied by distinct inflammatory characteristics, which induce the formation of pulmonary lesions. According to previous reports, adoptive transfer of Vγ2Vδ2 T cells in a *M. tuberculosis*-infected macaque model significantly attenuated TB lesions (11), suggesting the efficacy of Vγ2Vδ2 T-cell transfer in controlling inflammation. The inhibitory effect of γδ T cells on inflammation was also reported in a mouse model (41). Our clinical observations of all eight patients with MDR-TB suggested the inhibitory effect of Vγ9Vδ2 T cells on *M. tuberculosis*-induced pulmonary inflammation/lesions regardless of being combined with anti-TB drugs or administered alone. Accordingly, we demonstrated that allogeneic Vγ9Vδ2 T-cell-based immunotherapy was efficient in relieving pulmonary lesions.

Given that the immune function of patients with TB is generally impaired, and CD4 and CD8 T cells have an important role in controlling TB infection (42, 43), an evaluation of the functional phenotypes of circulating immune cells is needed in the context of Vγ9Vδ2 T-cell therapy. By analyzing the functional phenotypes of immune cell data in eight patients before and after Vγ9Vδ2 T-cell therapy, we discovered that the proportion of immature NK cells was significantly reduced, and the proportion of mature NK cells was significantly increased. There was no visible change in the proportion of other cells. The results implied that allogeneic Vγ9Vδ2 T cells enhanced NK cell functions of patients with MDR-TB.

In parallel, we also analyzed the functional phenotypes in the immune cell data of patient #8 who only received limited courses (4) of allogeneic Vγ9Vδ2 T-cell therapy. We discovered that the populations of functional subsets of CD4 and CD8 T cells, such as effective memory CD4^+^ T cells, effective memory CD8^+^ T cells, helper T cells, and killer T cells, were increased after adoptive transfer of allogeneic Vγ9Vδ2 T cells. In addition, the PD-1 expression of CD8 T cells was significantly reduced, further endorsing our assumption that PD-1^+^CD8^+^ T cells are favorable factors for *M. tuberculosis* infection (44). Intriguingly, we also found that the NK cell subsets were dramatically increased after adoptive transfer of allogeneic Vγ9Vδ2 T cells, further indicating the beneficial effect of cell therapy for patients with TB, as patients with pulmonary tuberculosis show a reduced frequency of NK cell subsets (45). There has always been some controversy on the role of humoral immunity in TB. The contribution of B cells and antibodies in the protection against *M. tuberculosis* infection remains unclear (46). Here, we also observed no great changes in the populations of B cells before and after cell therapy. Altogether, our study indicated a certain regulatory effect of Vγ9Vδ2 T cells on cellular immunity.

Body weight was also used as an observational indicator though not a significant indicator during treatment of TB. We noticed that the body weight of half of the patients steadily increased, whereas the other half lost weight. Due to the low number of patients and the variation in their drugging regimens and nutritional status, we could not determine whether these changes in body weight were associated with Vγ9Vδ2 T-cell therapy. An increasing number of studies have shown that host lipid metabolism is involved in antituberculosis immunoregulation. For example, *M. tuberculosis* has been shown to impair mitochondrial fatty acid oxidation through the miR-33 host miRNA (47). A previous study in *M. tuberculosis*-infected macaques noted that adoptive transfer therapy of Vγ2Vδ2 T cells stabilized the body weight of macaques (11), implying that γδ T-cell-based immunotherapy regulated the host metabolism. Interestingly, we noted that the body weight of patient #8, who received four courses of allogeneic Vγ9Vδ2 T-cell therapy alone, was increased in a stepwise manner. Moreover, we also found that plasma carnitine concentration was decreased. Carnitine is known to not only promote lipid metabolism but also reduce the levels of triglycerides, total cholesterol, and LDL-cholesterol (48). Although these data were derived from only a single patient, it suggested that allogenic Vγ9Vδ2 T cells might favor antituberculosis treatment through the regulation of lipid metabolism. More studies will be needed in the future to support this observation.

Microbiota have been found to be closely related to the occurrence and development of many diseases (49–52). For instance, dysbiosis of intestinal microbiota aggravated pulmonary *M. tuberculosis* infection in a mouse model, resulting in increased *M. tuberculosis* extrapulmonary transmission (19). In this study, we only analyzed one precell therapy sample and one postcell therapy sample from patient #8 using 16S rRNA gene sequencing. We found that the population of commensal bacteria or probiotics of the patient was increased, whereas the population of pathogenic bacteria was decreased. This result suggested that allogeneic Vγ9Vδ2 T-cell therapy alters the constitution of intestinal microbiota in patients with MDR-TB. However, these findings need further investigation in future clinical trials.

*Mycobacterium tuberculosis* load is an important determinant for the evaluation of therapeutic efficacy. We also monitored changes in *M. tuberculosis* sputum using both sputum smear acid-fast bacillus and sputum culture testing. At the completion of the treatment period, the sputum *M. tuberculosis* of seven patients was detected to be negative in three consecutive readings. However, the sputum *M. tuberculosis* of patient #2, whose disease was very severe, remained positive at the end of Vγ9Vδ2 T-cell treatment. Clinically, the cure prevalence proportions were 54% for MDR-TB patients. Strikingly, in our study cohort, the cure prevalence proportions were 87.5% for MDR-TB patients who were treated with Vγ9Vδ2 T cells plus anti-MDR-TB drugs. In contrast, the therapeutic efficacy of Vγ9Vδ2 T cells plus anti-MDR-TB drugs was notably higher than anti-MDR-TB drugs only. We considered that using Vγ9Vδ2 T-cell therapy could alleviate the *M. tuberculosis* load *in vivo*.

In conclusion, this clinical trial creatively applied allogenic Vγ9Vδ2 T cells for the treatment of patients with MDR-TB and clearly demonstrated that allogenic Vγ9Vδ2 T-cell infusion is safe in the clinical setting. Based on the results of the CT images of the patient, the assessment of peripheral immune cell function, and sputum *M. tuberculosis* detection, we believe that Vγ9Vδ2 T-cell therapy is effective. Taken together, this trial provided a new direction for biotherapy of MDR-TB and other hard-to-treat infectious diseases, such as TB/HIV and TB/HBV infections. However, due to the limitations of single-arm studies, the number of patients enrolled was limited. In the future, we aim to design a rigorous and standardized randomized controlled clinical trial to systematically and multidimensionally evaluate the therapeutic efficacy of Vγ9Vδ2 T cells.

## Data Availability Statement

The datasets presented in this study can be found in online repositories. The names of the repository/repositories and accession number(s) can be found below: NCBI BioProject and PRJNA776411.

## Ethics Statement

The Regional Ethics Committee of Shenzhen Third People’s Hospital approved the study protocol (Approval ID SZLY2018017). The patients/participants provided their written informed consent to participate in this study. Written informed consent was obtained from the individual(s) for the publication of any potentially identifiable images or data included in this article.

## Author Contributions

Protocol design: ZY, YZW, GZ, and GD. Cell expansion and quality control: JLiang, ML, and YZW. Clinical therapy for patients: GD and LF. Immuno-function testing and statistics analysis: JLiang, LF, YZW, ZY and GZ. Patient recruitment: GD, JLiang, YZW, LF, ZY, SL, PZ, ZW, and LLiu. Patient follow-up: JLiang, YZW, ML, FL, GD and GZ. Manuscript writing and revision: YZW, JL, ZY. Other co-authors contributed to either scientific inputs/comments or patient follow-up for this long-term clinical trial project.

## Funding

This work was supported by the National Key Research and Development Plan (No. 2019YFC0840602, 2020YFA0907201), the Guangzhou Science and Technology Planning Project (No. 201604020006), the National Science and Technology Major Project for Control and Prevention of Major Infectious Diseases of China (No.2018ZX10715004), and the National Natural Science Foundation of China (No. 81873958, 81902031, 82170009), Guangdong Scientific and Technological Foundation (No. 2019B1515120041, 2019B1515120067, 2020B1111170014, 2020A1515010977), Shenzhen Scientific and Technological Project (No.KCXFZ202002011007083), the Scientific Foundation of Shenzhen Municipal Health Commission (No. SZLY2018017), the Sanming Project of Medicine in Shenzhen (No. SZSM201911009), the “Dengfeng Plan” High-level Hospital Construction Opening Project of Foshan Fourth People’s Hospital (No. FSSYKF-2020016), and the Foshan Scientific and Technological Innovation Project (No. FS0AAKJ218-1301-0035).

## Conflict of Interest

The authors ML and LiL were employed by Guangdong GD Kongming Biotech Ltd.

The remaining authors declare that the research was conducted in the absence of any commercial or financial relationships that could be construed as a potential conflict of interest.

## Publisher’s Note

All claims expressed in this article are solely those of the authors and do not necessarily represent those of their affiliated organizations, or those of the publisher, the editors and the reviewers. Any product that may be evaluated in this article, or claim that may be made by its manufacturer, is not guaranteed or endorsed by the publisher.
