# Supplementary material for: Allogeneic Vγ9Vδ2 T-Cell Therapy Promotes Pulmonary Lesion Repair: An Open-Label, Single-Arm Pilot Study in Patients With Multidrug-Resistant Tuberculosis
Source: Front Immunol. 2021 Dec 15;12:756495. doi: 10.3389/fimmu.2021.756495 (PMC8715986; doi:10.3389/fimmu.2021.756495)
Supplement: Supplementary file 1 [file Presentation_1.pptx]

## Slide 1
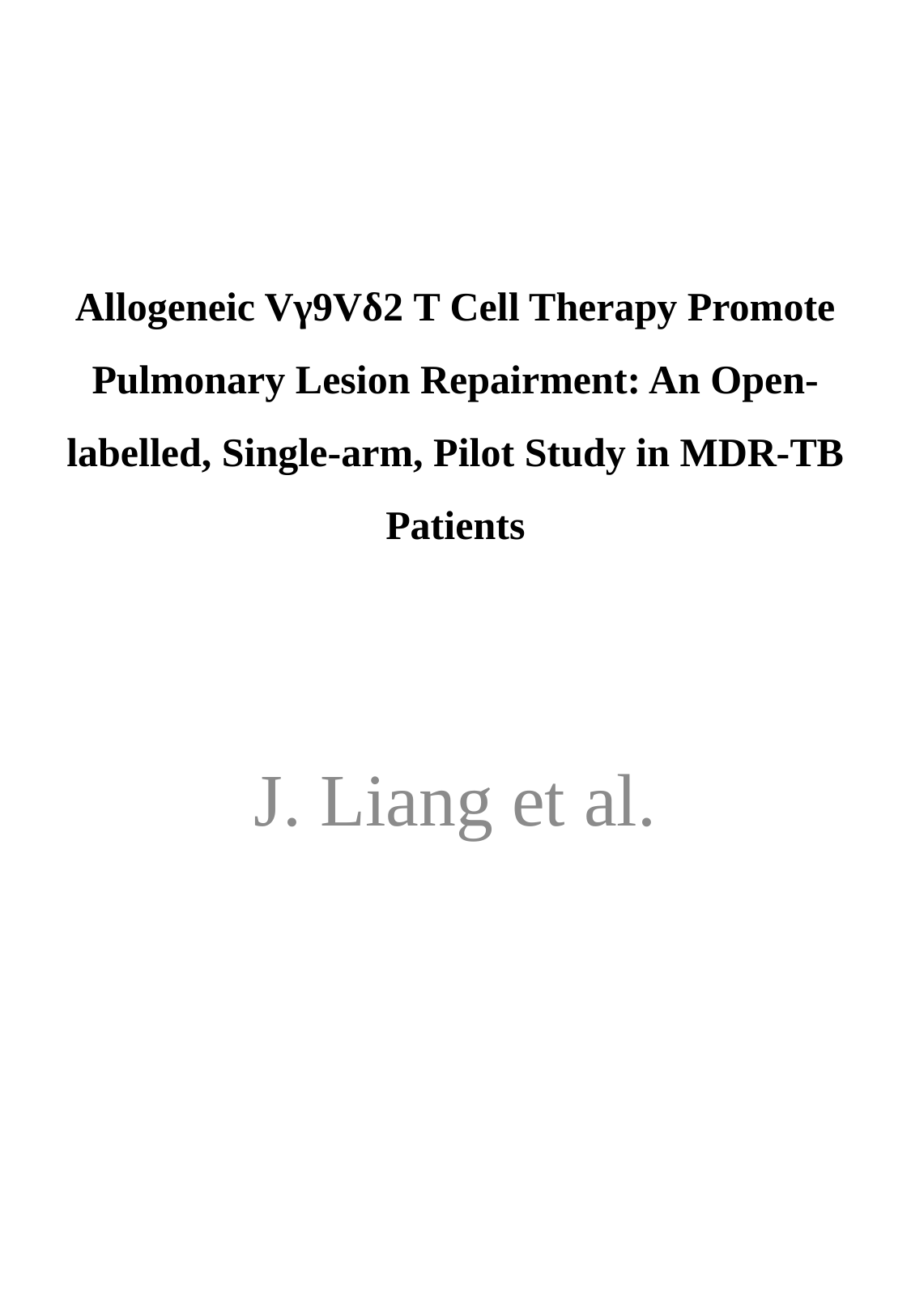

# Allogeneic Vγ9Vδ2 T Cell Therapy Promote Pulmonary Lesion Repairment: An Open-labelled, Single-arm, Pilot Study in MDR-TB Patients
J. Liang et al.

## Slide 2
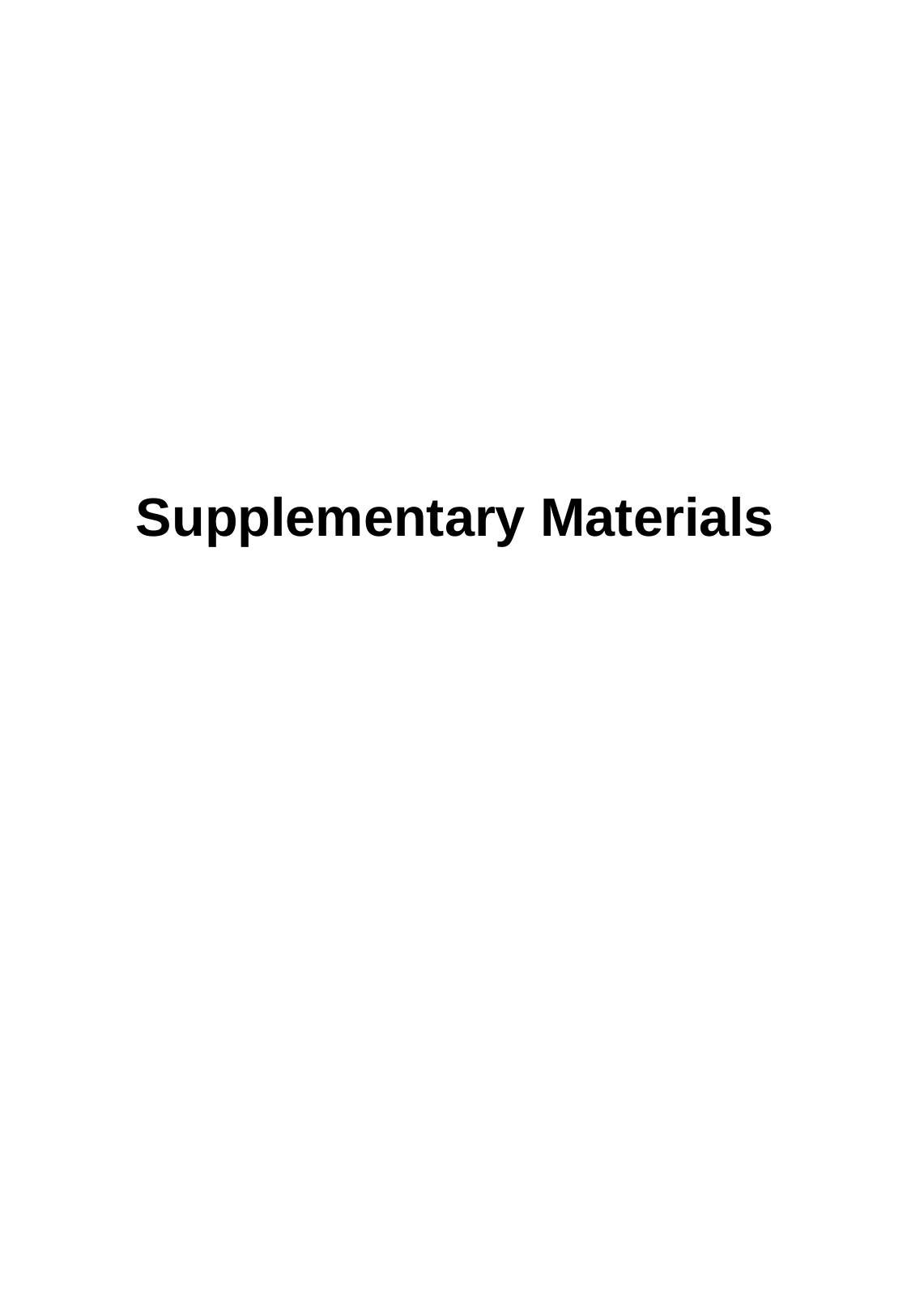

Supplementary Materials

## Slide 3
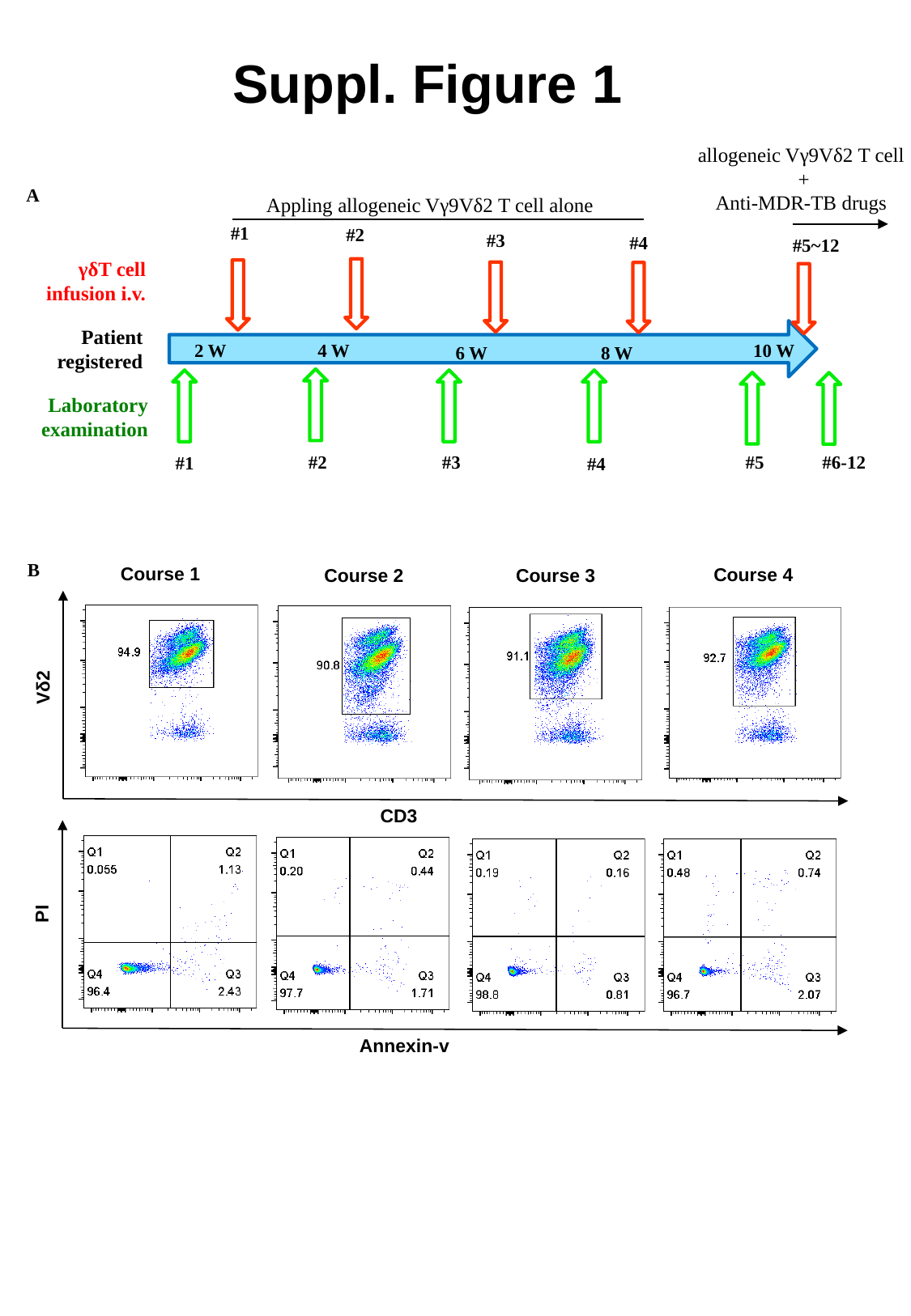

Suppl. Figure 1
allogeneic Vγ9Vδ2 T cell
+
Anti-MDR-TB drugs
A
Appling allogeneic Vγ9Vδ2 T cell alone
#1
#2
#3
#4
#5~12
γδT cell infusion i.v.
 Patient
registered
2 W
4 W
10 W
6 W
8 W
Laboratory examination
#2
#5
#3
#6-12
#1
#4
B
Course 1
Course 4
Course 2
Course 3
Vδ2
CD3
PI
Annexin-v

## Slide 4
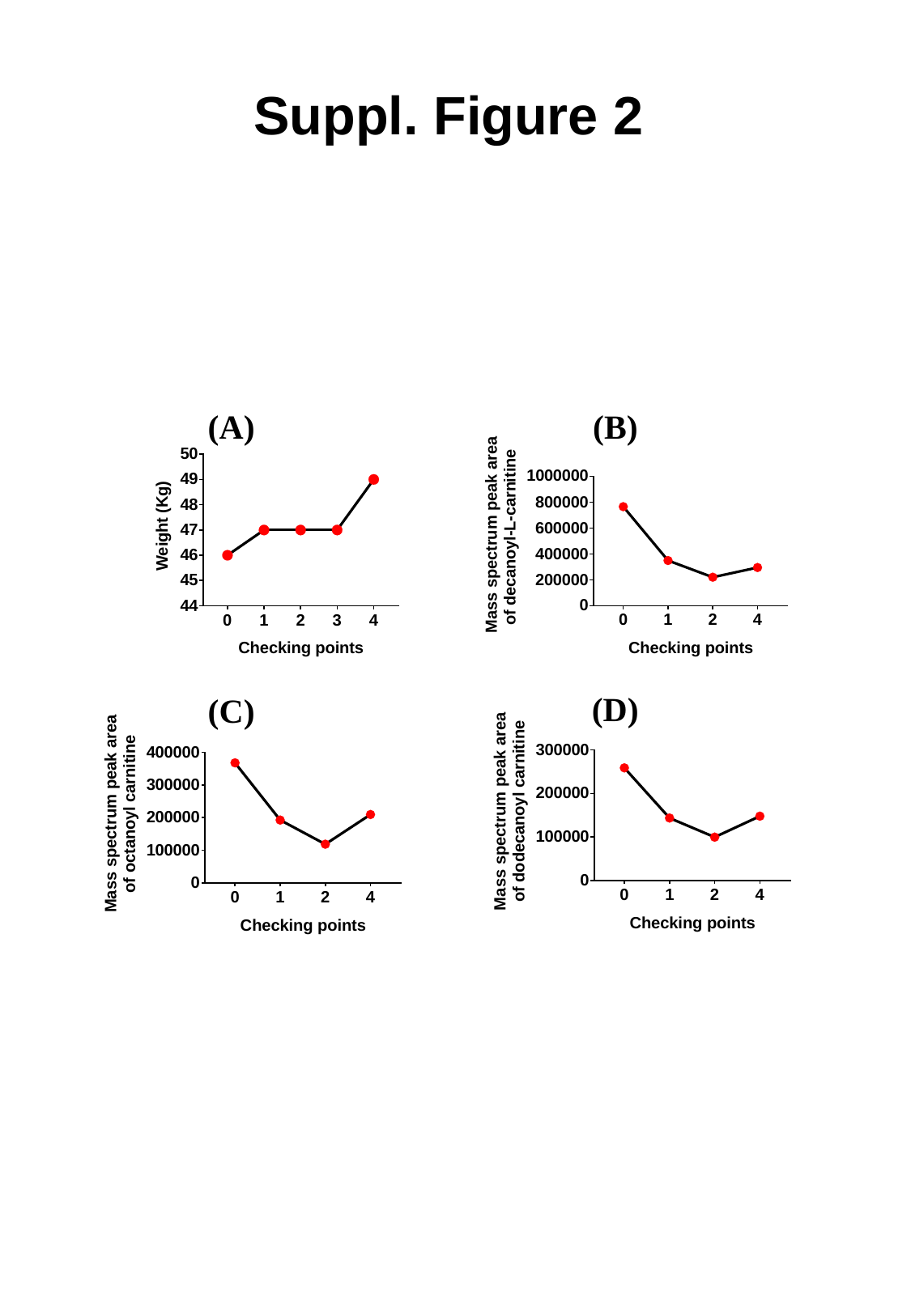

Suppl. Figure 2
(A)
(B)
(D)
(C)

## Slide 5
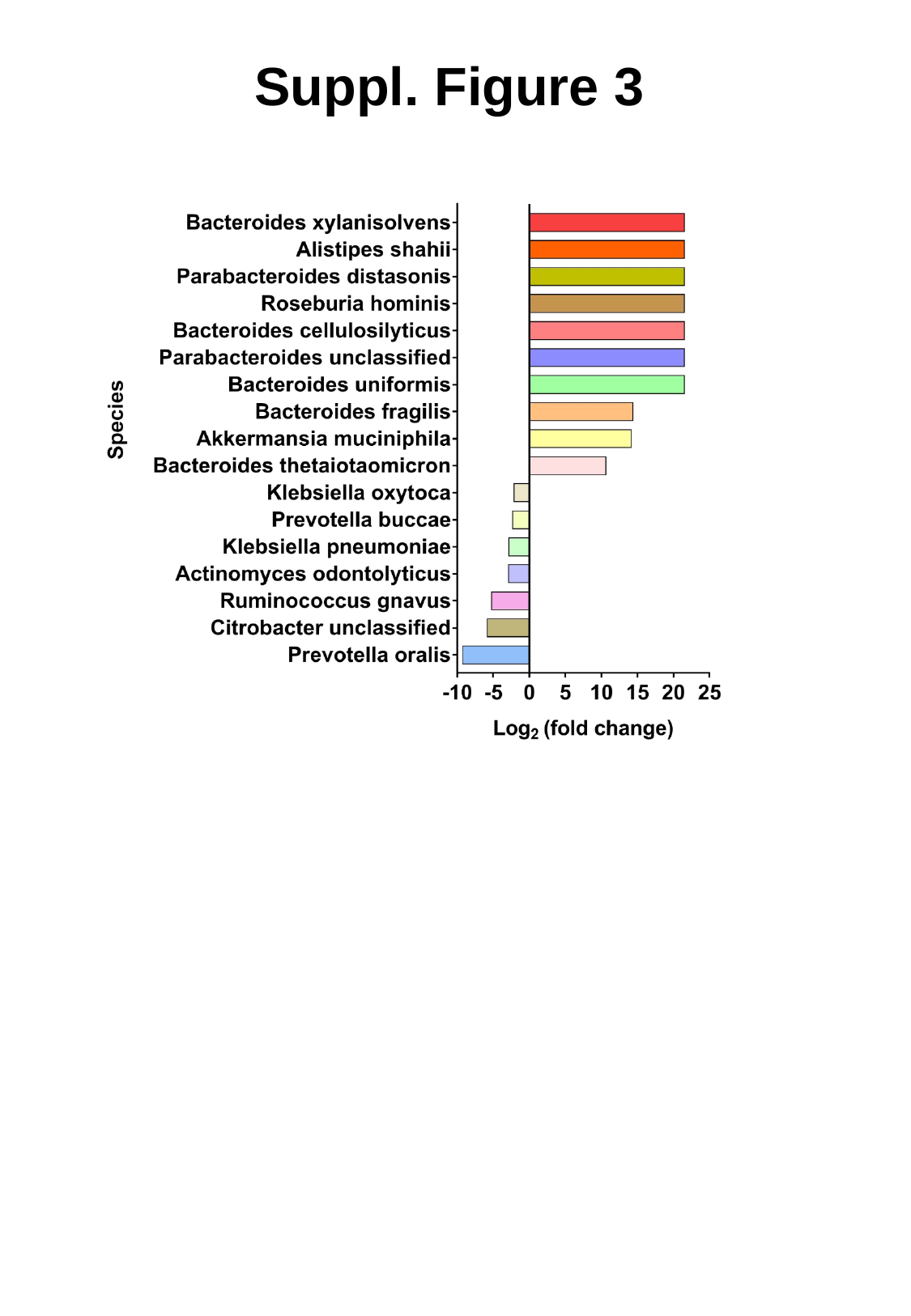

Suppl. Figure 3

## Slide 6
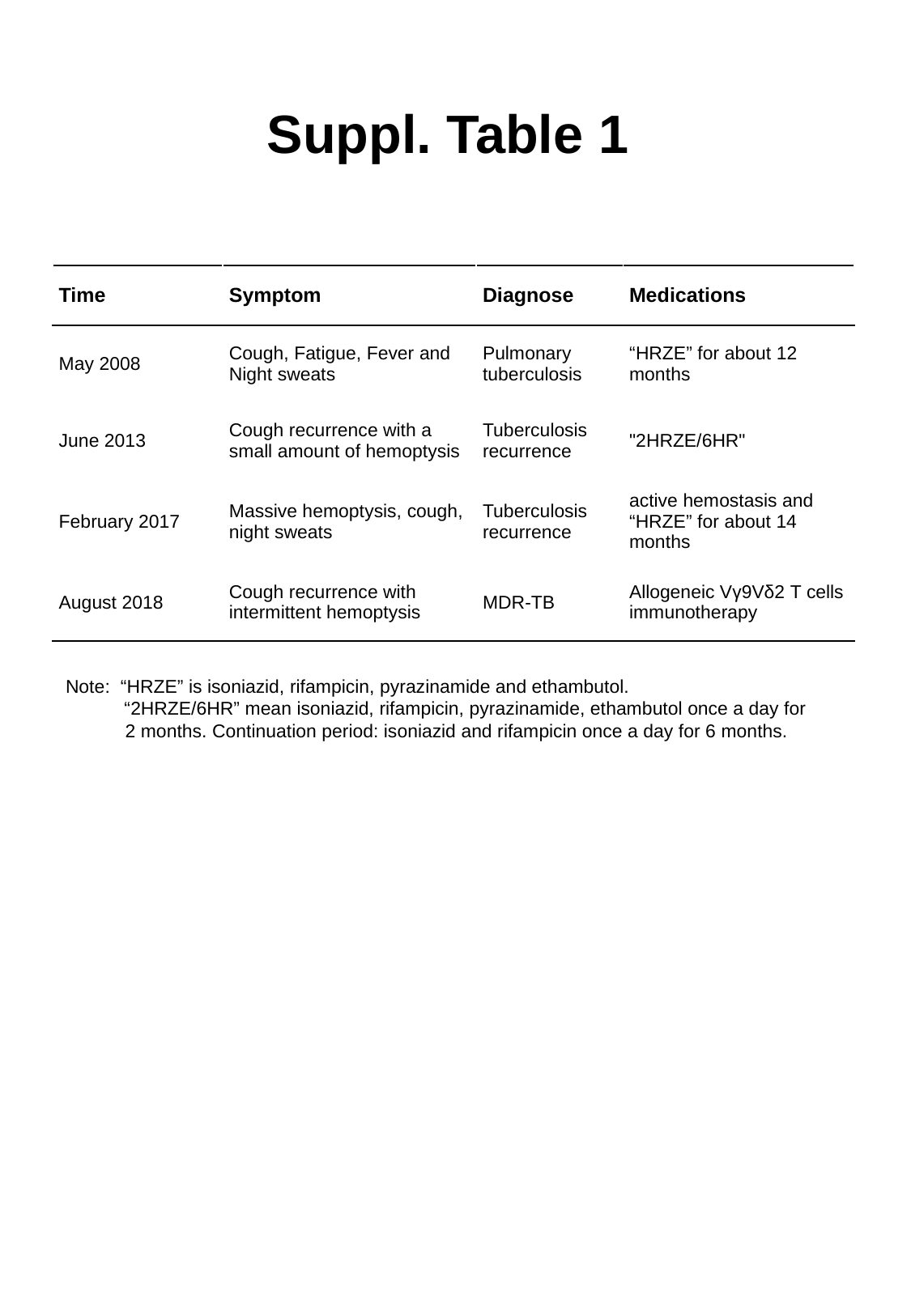

Suppl. Table 1
| Time | Symptom | Diagnose | Medications |
| --- | --- | --- | --- |
| May 2008 | Cough, Fatigue, Fever and Night sweats | Pulmonary tuberculosis | “HRZE” for about 12 months |
| June 2013 | Cough recurrence with a small amount of hemoptysis | Tuberculosis recurrence | "2HRZE/6HR" |
| February 2017 | Massive hemoptysis, cough, night sweats | Tuberculosis recurrence | active hemostasis and “HRZE” for about 14 months |
| August 2018 | Cough recurrence with intermittent hemoptysis | MDR-TB | Allogeneic Vγ9Vδ2 T cells immunotherapy |
Note: “HRZE” is isoniazid, rifampicin, pyrazinamide and ethambutol.
 “2HRZE/6HR” mean isoniazid, rifampicin, pyrazinamide, ethambutol once a day for 2 months. Continuation period: isoniazid and rifampicin once a day for 6 months.
